# Attentional Load Effects on Beta Oscillations in Healthy and Schizophrenic Individuals

**DOI:** 10.3389/fpsyt.2015.00149

**Published:** 2015-10-19

**Authors:** Shahab Ghorashi, Kevin M. Spencer

**Affiliations:** ^1^Research Service, Veterans Affairs Boston Healthcare System, Boston, MA, USA; ^2^Department of Psychiatry, Harvard Medical School, Boston, MA, USA

**Keywords:** schizophrenia, electroencephalogram, gamma oscillation, beta oscillation, attention

## Abstract

Attentional deficits are prominent among the cognitive disturbances found in schizophrenia. Given that schizophrenia is also characterized by abnormalities in high-frequency oscillations, we investigated whether attentional function in schizophrenia is related to abnormalities in high-frequency oscillations in a visual discrimination task in which attentional load was manipulated. Sixteen healthy control subjects (HC) and 23 chronic schizophrenia patients (SZ) discriminated between target discs (*p* = 0.2) and standard discs (*p* = 0.8). Attentional load was manipulated by varying the size difference between the target and standard discs across blocks: large (Easy condition), medium (Medium), and small (Difficult). The electroencephalogram was recorded and the oscillations evoked by the standard stimuli were analyzed using the Morlet wavelet transform. Subjects’ performance decreased as attentional load increased, but HC and SZ did not differ. Attentional load increased β phase-locking factor at frontal, parietal, and occipital electrode sites in HC but not SZ. In SZ, however, there was a correlation between the β attentional load effect and overall *d*′, indicating that high-performing SZ had relatively normal β attentional load effects. These results show that variations in attentional load are associated with β oscillations and provide a link between attentional dysfunction and β-generating neural circuitry in schizophrenia.

## Introduction

A growing body of evidence implicates high-frequency oscillatory activity in the electroencephalogram (EEG) in various aspects of attention. Studies of animals [e.g., Ref. (1–3)] and humans [e.g., Ref. (4–6)] have shown that attention is associated with enhanced β (13–30 Hz) and γ (30–100 Hz) band oscillations. These high-frequency oscillations also appear to be involved in the control of attention, possibly coding templates of attended features in attentional control areas and transmitting bias signals from control areas to sensory areas via long-distance synchronization (7–9).

Attention deficits are prominent among the cognitive disturbances that are typically found in individuals with schizophrenia (10, 11). Schizophrenia is also characterized by abnormalities in high-frequency oscillations associated with both sensory/perceptual processing [e.g., Ref. (12–20)] and cognitive control processes [e.g., Ref. (21–23)]. These abnormalities have been proposed to originate in disturbances of cortical microcircuitry, such as in recurrent inhibition from fast-spiking, parvalbumin-expressing interneurons to pyramidal cells (24). One question that has not yet been addressed is whether attention deficits in schizophrenia are related to abnormalities in high-frequency oscillations. Here, we tested this hypothesis by examining how oscillatory activity in chronic schizophrenia patients (SZ) and matched healthy control subjects (HC) was affected by varying the attentional load of a simple visual discrimination task [cf. Ref. (25)]. Subjects performed a visual oddball task in which they discriminated between standard stimuli of a constant size and target stimuli that varied in size across blocks. As the target/standard discrimination became more difficult, the attentional load of the task increased.

## Materials and Methods

### Subjects

This study was approved by the Institutional Review Boards of the Veterans Affairs Boston Healthcare System and Harvard Medical School. Written informed consent was obtained from the subjects after the study was described to them. All subjects were paid for their participation in the study.

Subjects were 16 HC (two female) and 23 SZ (one female). SZ were recruited from outpatient clinics at the Veterans Affairs Boston Healthcare System. SZ were diagnosed based on the Structured Clinical Interview for DSM-IV [SCID (26)] and medical record review. HC were recruited from the Boston metropolitan area and matched the SZ at the group level on age, handedness (27), parental socioeconomic status [PSES (28)], gender proportion, and estimated premorbid intelligence, as assessed by performance on the Reading scale of Wide Range Achievement Test [WRAT-3 (29)]. See Table 1 for demographic and clinical characteristics. Clinical symptoms were assessed using the Scale for the Assessment of Positive Symptoms [SAPS (30)] and the Scale for the Assessment of Negative Symptoms [SANS (31)]. Medication dosage in chlorpromazine equivalents was calculated using the conversion factors of Stoll (32) and Woods (33).

**Table 1:** **Demographic and clinical data and between-group comparisons for the healthy control (HC) and schizophrenia patient (SZ) groups**.

	HC (*N* **=** 16)	SZ (*N* **=** 23)	Statistic	*p*
Age (years)	41.3 ± 5.0	42 ± 9.6	*t*_(37)_ = −0.26	0.81
Parental socioeconomic status	2.6 ± 1.1	2.6 ± 1.0	*t*_(37)_ = 0.05	0.96
WRAT-3	49.31 ± 5.52	47.64 ± 4.61	*t*_(36)_ = 0.988	0.33
Age of onset (years)		24.3 ± 6.6		
Positive symptom total (SAPS)		9.4 ± 3.4		
Negative symptom total (SANS)		10.5 ± 6.2		
Medication dosage (chlorpromazine equivalent)		365.8 ± 379.6		
		Range: 100–1467		

*Mean ± SD are given for each variable*.

*WRAT-3, Wide Range Achievement Test; SAPS, Scale for the Assessment of Positive Symptoms; SANS, Scale for the Assessment of Negative Symptoms*.

Exclusion criteria for all subjects were (1) left-handedness, (2) history of electroconvulsive shock therapy, (3) history of neurological illness including epilepsy, (4) lifetime history of substance dependence or history of substance abuse within the past 5 years, (5) history of steroid use, and (6) estimated premorbid intelligence quotient (WRAT-3 score) below 75. Additional exclusion criteria for HC were the presence of an Axis-I disorder [from the SCID-Non-Patient edition (34)], and having a first-degree relative with an Axis I disorder.

### Stimuli and Procedure

To study the effects of attentional load, we used an oddball task in which the difficulty of discriminating target from standard stimuli was varied across blocks (Easy, Medium, and Difficult conditions). Targets and standards differed in size, and the size of the targets was varied while the size of the standards was kept constant. As the responses to standards provide measures of brain activity that do not include motor- or deviance-related activity (as do the responses to targets), attentional load should be the only factor that would affect the responses to standards. On each trial, subjects classify the stimulus as a standard or a target, which involves allocating attentional resources to the comparison of the stimulus percept with templates of the targets and standards in working memory. As the comparison becomes more difficult (the size of standards and targets becomes more similar), more attentional resources must be allocated to the comparison process.

**Figure 1:**
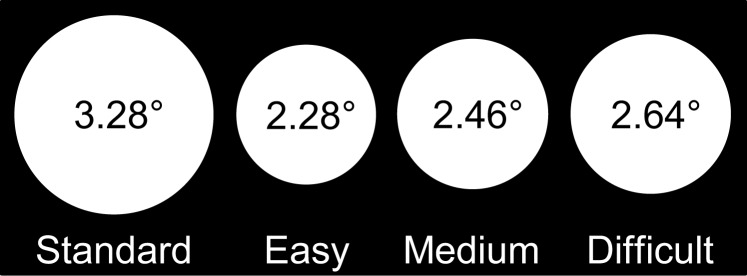
**Stimuli in each condition of the experiment and their sizes in degrees of visual angle: the standard disc (left) and target discs in the Easy, Medium, and Difficult conditions**.

Stimuli were white discs presented on a black background at the center of the screen. The diameter of the standard discs was 3.28° of visual angle, and the diameters of the target discs in the Easy, Medium, and Difficult conditions were 2.28°, 2.46°, and 2.64° of visual angle, respectively (Figure 1).

Subjects performed three blocks of 180 trials each: Easy (the greatest size difference between the target and the standard discs), Medium, and Difficult (the smallest size difference between the target and the standard discs). On 144 (80%) of those trials, the standard disc was displayed and on the rest of the trials (20%) the target was displayed. Standard and target trials were presented in pseudorandom order. The order of the blocks was counterbalanced across subjects. Each block of trials was preceded with practice trials to familiarize the subjects with the difficulty of the required target/standard discrimination.

Subjects were seated at a distance of 1 m from the monitor (nasion to the central fixation point). Stimuli were presented for 82 ms with an inter-stimulus interval of 1058 ms (onset-to-onset). Subjects were instructed to respond only to the targets by pressing a key on the response pad with their right hand as quickly and accurately as possible.

### EEG Acquisition and Analysis

The EEG was recorded with a Biosemi ActiveTwo system using active electrodes in an electrode cap at 71 standard EEG and electro-oculogram (EOG) sites (DC–100 Hz bandpass filter, 512 Hz digitization rate). The DC offsets were kept below 25 mV. During data acquisition, all channels were referred to the system’s internal loop (CMS/DRL sensors located in the parietal region) and off-line re-referenced to the left mastoid electrode. The bipolar vertical EOG was derived from electrode Fp1 and an electrode below the left eye. The horizontal EOG was derived from electrodes on the left and right outer canthi.

For each of the 540 trials presented to each subject, a 1000-ms epoch was extracted from 500 ms pre-stimulus to 498 ms post-stimulus using BrainVision Analyzer 1.0 (Brain Products GmbH). Further processing was performed using software in MATLAB (Mathworks, Inc.) and IDL (Exelis Visual Information Solutions, Inc.). Error trials were excluded from processing, and an initial artifact detection scan was run. The artifact exclusion criteria were (1) >±90 μV change in one time point and (2) amplitude range within an epoch exceeding 200 μV. Then independent component analysis [implemented in the *runica.m* program from EEGLAB (35)] was used to remove ocular and muscle artifacts. Independent components representing artifacts were identified based on their characteristic topographic, temporal, and spectral signatures (36–38). Next, a second artifact detection scan was run. Finally, the retained correct-response, artifact-free epochs were re-referenced to the average reference (39), computed on all 68 scalp channels, excluding the EOG channels. The number of epochs retained per subject was (mean ± SD) 493 ± 31 for HC and 485 ± 47 for SZ, and these numbers did not differ [*t*_(37)_ = 0.596, *p* = 0.56]. None of the subjects had more than 1/3 of trials per condition rejected.

EEG analyses focused on the responses to standard stimuli, which were physically identical in each condition and not influenced by target- or response-related processing. Event-related potentials (ERPs) and spectral measures were computed from the artifact-free single-trial epochs. Time-frequency decomposition was performed using the Morlet wavelet transform (frequency/duration ratio *f*_0_/σ*_f_* = 6), applied in 1 Hz steps from 4 to 100 Hz at each time point to yield time-frequency (TF) maps of phase-locking factor (PLF) values (40). PLF is computed as one minus the circular variance of phase (at each time point and wavelet frequency) across the set of single trials in a condition for each subject. This measure reflects the degree to which a set of signals match in phase, or are phase-locked, relative to a reference time point (such as stimulus onset or RT). PLF values range from 0 (no synchrony, random distribution of phases) to 1 (perfect synchrony, same phase on every trial). (We also measured evoked power but do not report it here, as it yielded a very similar but weaker pattern of results as PLF. Analyses of total power did not reveal any effects of group or attentional load.) Average pre-stimulus baseline values from −100 to 0 ms were subtracted from the PLF TF maps.

### Statistical Analyses

Statistical non-parametric mapping (SnPM) was used to find clusters of TF elements (time points at each frequency) in which there was a significant interaction between the factors Group (HC/SZ) and Difficulty (Easy/Difficult). This approach, based on the permutation test, has several advantages over parametric statistical tests (41, 42), particularly that it does not rely upon assumptions about the statistical distribution of the data. Thus, the SnPM approach is more sensitive than parametric tests when the assumptions underlying the latter are not met (e.g., normality), which is likely for the PLF measure. Additionally, the permutation test provides control for multiple comparisons, since all the TF elements are permuted in parallel. In practice, we found it necessary to apply additional criteria to control for multiple comparisons. Our SnPM approach consisted of the ­following steps:

(1) TF *t*-maps were computed by performing *t*-tests on each TF element across the epoch for each channel. The Group × Difficulty interaction map was computed with between-groups *t*-tests on the Difficult minus Easy difference maps, which is equivalent to a 2 × 2 ANOVA design.
(2) TF maps of *p* values were computed for each *t*-map using the permutation test (α = 0.05, two-tailed, 1000 permutations). A difference map (Difficult minus Easy) was computed for each subject, and the assignment of subjects to the groups was shuffled on each permutation. The *p* value of each TF element was obtained by determining the percentile rank of the observed *t* value in the shuffled *t* distribution.
(3) The resulting *p*-maps were thresholded at *p* > 0.975 for positive interactions (HC difference > SZ difference) and *p* < 0.025 for negative interactions (SZ difference > HC difference). TF elements with *p* values above/below these thresholds were retained only if they were part of a cluster with a duration of at least one cycle of the respective frequency (e.g., 25 ms for a 40 Hz cluster).
(4) The thresholded *p*-maps were summed across the scalp EEG channels (*N* = 68) to create a channel sum histogram of TF clusters. This histogram represents the number of channels on which each TF cluster was found. The channel sum histogram was then thresholded at the 95th percentile of the distribution for that histogram, retaining only clusters in which the number of contributing channels was at the upper 5% of the distribution. The reasoning for this step was that since a large number of clusters occurred at only one electrode, “true” effects should be present on multiple channels due to volume conduction.
(5) A one-cycle duration cutoff was applied again to the channel sum histogram, so that all the final TF clusters were at least one cycle in duration at their respective frequencies. The electrodes contributing to each cluster were plotted in topographic maps with color codes indicating the percentage of the cluster area to which the electrode contributed.

Task performance was measured with error rate, median reaction time (RT), and the signal detection measure *d*′ (discriminability), which measures subjects’ ability to discriminate between stimulus classes independently of biases to respond to one class over the other (43). PLF of visual-evoked γ oscillations was measured at electrodes and latency windows determined from the grand averages. Performance measures and PLF were analyzed with analysis of variance (ANOVA) in the design Group (HC/SZ) × Difficulty (Easy/Medium/Difficult) [×Hemisphere (Left/Right) × Electrode factors, where relevant]. The Greenhouse–Geisser correction for inhomogeneity of variance (44) was applied for factors with more than two levels and is reflected in the reported *p* values. Correlation analyses employed the non-parametric Spearman’s ρ (two-tailed). For all statistical analyses, α = 0.05.

## Results

### Task Performance

**Figure 2:**
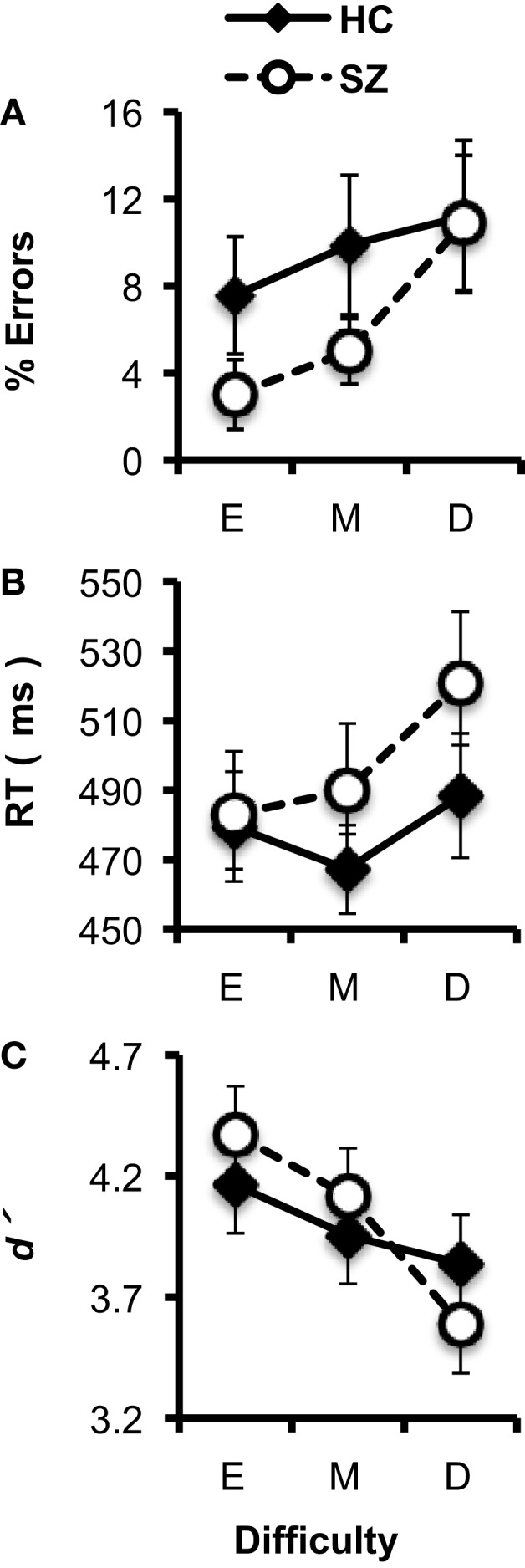
**Task performance data (error bars indicate SE) for the healthy control (HC) and schizophrenia patient (SZ) groups in the three experimental conditions (E: Easy; M; Medium; D: Difficult)**. **(A)** Error rates. **(B)** Median reaction time (RT). **(C)***d*′.

In general, subjects’ performance decreased as the difficulty of the target/standard discrimination increased (Figures 2A–C). Subjects’ error rates increased with Difficulty (*F*_2,74_ = 5.61, *p* < 0.05). The HC and SZ groups were not significantly different in overall error rate (*F*_1,37_ = 1.09, *p* = 0.30), and the Group × Difficulty interaction was not significant (*F*_2,74_ = 1.09, *p* = 0.33). Subjects’ median RTs also increased with Difficulty (*F*_2,74_ = 5.77, *p* < 0.01), but the two groups did not have significantly different overall RTs (*F*_1,37_ < 1, *ns*), and the Group × Difficulty interaction (*F*_2,74_ = 1.54, *p* = 0.22) was not significant.

Analyses of *d*′ indicated that overall, subjects’ discrimination between targets and standards decreased as Difficulty increased (*F*_2,74_ = 12.49, *p* < 0.001). HC and SZ did not differ significantly in overall *d*′ (*F*_1,37_ < 1, *ns*), and there was no significant Group × Difficulty interaction (*F*_2,74_ = 2.56, *p* = 0.09). Thus, as the size of the target stimulus approached the size of the standard stimulus, the difficulty of the target/standard discrimination increased for both groups.

### Event-Related Potentials

**Figure 3:**
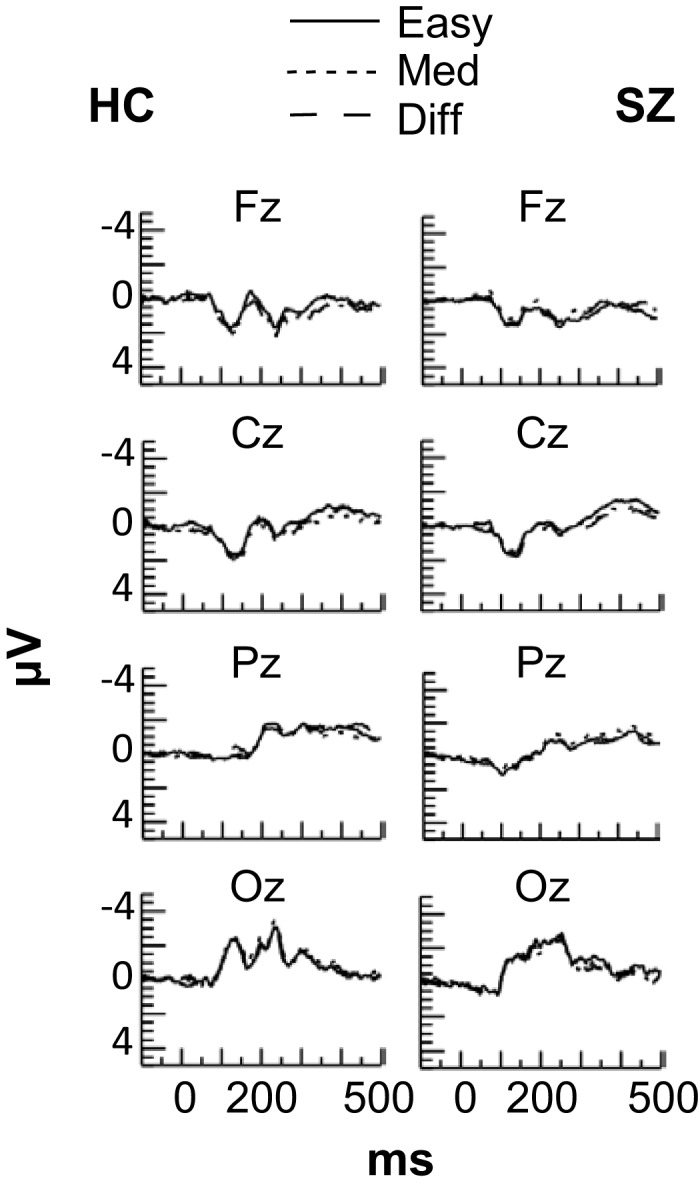
**Grand average event-related potentials at frontal (Fz), central (Cz), parietal (Pz), and occipital (Oz) electrode sites in each Difficulty condition for the HC and SZ groups**. No effects of Difficulty were observed for either group.

No effects of task difficulty were apparent in the ERPs for either subject group (see Figure 3), and the ERPs were not analyzed further.

### Oscillatory Activity

**Figure 4:**
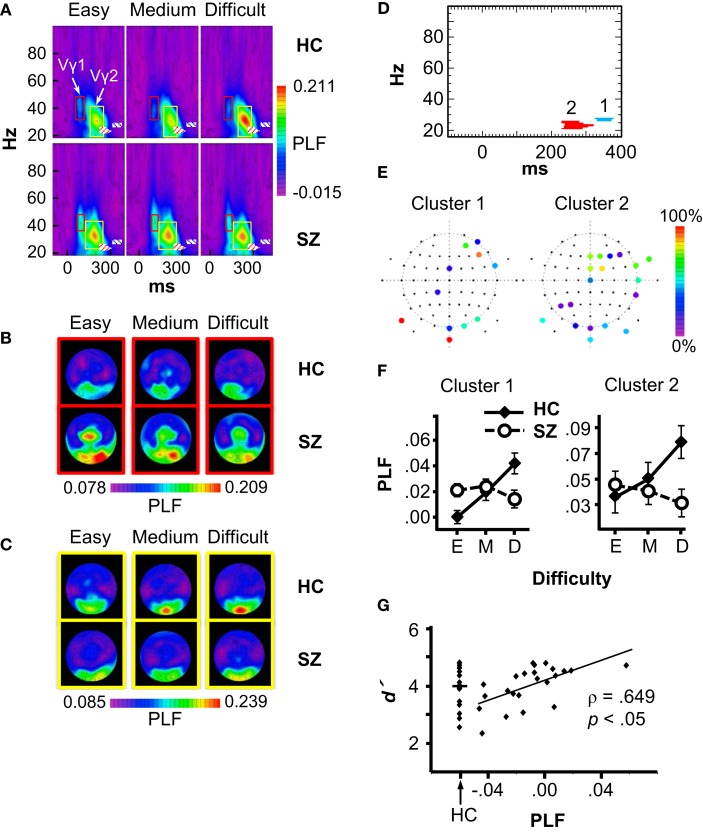
**Phase-locking factor (PLF) data**. **(A)** Grand average time-frequency (TF) PLF maps for the HC and SZ groups in each Difficulty condition. TF maps were averaged across all scalp electrodes. Boxes indicate the ranges in which the Vγ1 (red boxes) and Vγ2 (yellow boxes) oscillations were measured. Crosshatched boxes represent Cluster 1 (blue) and Cluster 2 (red) derived from statistical non-parametric mapping. **(B)** Topography of Vγ1 in each condition and group. **(C)** Topography of Vγ2 in each condition and group. **(D)** Statistical non-parametric mapping results showing the two Group × Difficulty PLF clusters (Cluster 1: 328–377 ms, 26–27 Hz; Cluster 2: 232–320 ms, 21–25 Hz). **(E)** Cluster topographies. The color scale indicates the percentage of the cluster to which each electrode contributed. **(F)** PLF values for each cluster in three difficulty conditions (E: Easy; M: Medium; D: Difficult). **(G)** Scatterplot of the correlation between the Cluster 1 Difficulty effect (Difficult minus Easy PLF) and overall *d*′ in the SZ group. The distribution of *d*′ values for HC is shown for comparison.

Statistical non-parametric mapping of the PLF data revealed two Group × Difficulty clusters, both with relatively long latencies and in the β frequency range (Figure 4D). Cluster 1 occurred at 328–377 ms in the 26–27 Hz frequency band, and had contributions mainly from frontal, occipital, and occipital-temporal electrodes (AF4, AF8, F6, FCz, FT8, CP1, P9, PO8, Oz, O2, and Iz) (Figure 4E, left). Using this cluster as a region of interest, we analyzed PLF in a full ANOVA with the design Group × Difficulty × Electrode. PLF increased with Difficulty in HC (*F*_2,30_ = 7.51, *p* < 0.01), but this effect was not significant in SZ (*F*_2,44_ = 2.52, *p* = 0.09), and these patterns differed between groups (Group × Difficulty: *F*_2,74_ = 11.43, *p* < 0.001) (see Figure 4F, left). The main effect of Difficulty was also significant (*F*_2,74_ = 4.24, *p* < 0.05).

Cluster 2 occurred at 232–320 ms and 21–25 Hz, with contributions from frontal, temporal, parietal, and occipital electrodes (Fz, F2, F4, F6, FCz, FC2, FT8, FT10, Cz, T8, TP8, P3, P5, P9, PO10, Oz, O1, O2, and Iz) (Figure 4E, right). Like Cluster 1, Cluster 2 PLF increased with Difficulty for HC (*F*_2,30_ = 12.09, *p* < 0.01), but this effect was not significant in SZ (SZ: *F*_2,44_ = 1.91, *p* = 0.16; Group × Difficulty: *F*_2,74_ = 12.43, *p* < 0.001) (see Figure 4F, right). The main effect of Difficulty approached significance (*F*_2,74_ = 3.03, *p* = 0.057). Neither of the β effects was correlated with medication dosage (Cluster 1: ρ = 0.12, *p* = 0.65; Cluster 2: ρ = −0.05, *p* = 0.85). Exploratory correlations between the β effects and clinical symptoms did not reveal any significant associations.

To determine whether the β attention effects were related to subjects’ task performance, we computed correlations between the PLF effect (Difficult minus Easy) for Clusters 1 and 2 (averaged over the electrodes contributing to each cluster) with task performance measures (Difficult minus Easy effects and overall means for RT, error rate, and *d*′). The *p* values of the correlations were Bonferroni-corrected (6 measures × 2 TF clusters × 2 subject groups). This analysis revealed (Figure 4G) a significant correlation for Cluster 1 in the SZ group between the PLF effect and overall *d*′ (ρ = 0.649, *p* < 0.05 corrected). The correlation was still significant after excluding one outlier subject (ρ = 0.63, *p* < 0.05 corrected). The range of the PLF effect varied from negative (Easy > Difficult) to positive (Difficult > Easy) across SZ. Thus, SZ who were better able to discriminate targets and standards in general had more normal (positive and larger) β attentional load effects.

Although effects of subject group and attentional load were not found on the stimulus-evoked γ oscillations in the statistical maps, since stimulus-evoked γ oscillations can be modulated by attention [e.g., Ref. (2, 5)], and visual γ deficits have been reported in schizophrenia (reviewed above), we analyzed the γ oscillations evoked by the standard stimuli to confirm these results. Standard stimuli evoked an early visual γ oscillation (Vγ1), which was measured at the occipital and occipito-temporal electrodes P9/10, PO7/8, PO9/10, and O1/2. The frequency range for Vγ1 was 33–48 Hz for HC and 36–48 Hz for SZ, and the time range was ~68–116 ms after stimulus onset for HC and ~80–116 ms for SZ (see Figure 4A red boxes, and Figure 4B). Vγ1 PLF did not differ between groups (*F*_1,37_ = 2.26, *p* = 0.14), nor was it modulated by task difficulty (*F*_2,74_ = 1.09, *p* = 0.34). The Group × Difficulty interaction was also not significant (*F*_2,74_ = 1.51, *p* = 0.23).

A later γ oscillation (Vγ2) with a similar scalp topography as Vγ1 was also observed, and was measured at the same electrodes as Vγ1. The frequency range for Vγ2 was 19–42 Hz for HC and 23–44 Hz for SZ, and the time range was ~146–269 ms after stimulus onset for HC and ~122–250 ms for SZ (Figure 4A yellow boxes, and Figure 4C). Vγ2 PLF was not significantly different between groups (*F*_1,37_ < 1, *ns*), nor was it modulated by task difficulty (*F*_2,74_ < 1, *ns*), and the Group × Difficulty interaction was not significant (*F*_2,74_ = 1.29, *p* = 0.28). Thus, the visual-evoked γ oscillations were not affected by attentional load, nor did they differ between subject groups.

## Discussion

We investigated how oscillatory activity in HC and SZ was affected by increasing the attentional load of a visual discrimination task. Increased attentional load in HC was manifested as increased stimulus-locked β activity at electrodes over cortical areas involved in attentional control and visual processing. In SZ, these β effects were not apparent at the group level, indicating an overall absence of attentional modulation of β. However, across SZ the later β effect was correlated with overall *d*′ values, such that those patients with a more normal late β effect were better able to discriminate targets from standards in general. It is important to note that SZ did not differ from HC in their overall ability to perform the task, as there were no group differences in error rate or RT. Therefore, the absence of attentional modulation of β activity in SZ at the group level cannot be attributed simply to a general deficit. Rather, the pattern of results suggests that attentional modulation of β activity is dysfunctional in schizophrenia overall but varies across individuals such that it is relatively preserved in SZ with better attentional function.

The β oscillation effects we observed were associated with changes in task difficulty, which modulated the attentional load of the task. As the β effects were found for the standard stimuli, they were unrelated to physical stimulus differences between conditions or manual response effects. Since the β effects occurred at relatively late latencies (232–377 ms), after the completion of several early stages of sensory and perceptual processing (i.e., those indexed by the C1, P1, and N1 ERP components and the Vγ1 oscillation), they are unlikely to reflect simple attentional modulation of sensory processes. While the inferences that can be drawn from scalp topographies regarding the neuroanatomical localization of EEG effects are limited, we note that the β effects were present at electrode sites over cortical areas involved in attentional control (frontal and parietal cortex), as well as visual cortex, and the right hemispheric lateralization of the β cluster topography is consistent with the right hemisphere’s predominant role in sustained attention [e.g., Ref. (45)]. One hypothesis regarding the functional significance of these oscillations is that they reflect attention-dependent processes by which a template of the target stimulus in short-term memory is compared with the current stimulus. This interpretation of the β effects is consistent with the hypothesis of Engel and Fries (46) that the role of β oscillations in top-down control is to maintain the current sensorimotor or cognitive state. β oscillations have been linked to cognitive domains, such as working memory [e.g., Ref. (47–49)], perceptual decision making [e.g., Ref. (50–53)], and attention (1, 3, 4, 7). Furthermore, computational modeling suggests that some β oscillations (although at lower frequency than found here) may be well suited for maintaining cell assembly states as required by working memory (54).

The finding that attentional modulation of β activity was dysfunctional in SZ suggests that neural circuit abnormalities in schizophrenia may extend to the infragranular layers of the cortex. There is evidence that β oscillations are generated in the deep layers of sensory and association cortex, while γ oscillations are generated in the granular and superficial layers [e.g., Ref. (55–57)]. Deficits of γ oscillations in schizophrenia have been hypothesized to be related to abnormalities in the function of inhibitory interneurons in the upper cortical layers, particularly the parvalbumin-expressing, fast-spiking class (24). The possible neural circuit abnormalities that could be responsible for the β deficit here are not as clear. The present β effects were found at electrodes lying over associational areas involved in attentional control, as well as visual cortex. Roopun et al. (57) found that β oscillations generated in layer 5 of sensory and association cortices arose from very different mechanisms, even though the frequency characteristics of those oscillations did not differ between areas. Sensory cortex β was generated by a circuit composed of pyramidal cells and electrically coupled low-threshold spiking interneurons. In contrast, association cortex β was generated by intrinsically bursting pyramidal cells in layer 5 that are synchronized through gap junctions.

The visual-evoked γ oscillations were not affected by attentional load (as with the ERPs), nor did they differ between SZ and HC. The literature on SZ deficits in visual γ oscillations has mixed findings. While some studies have reported reductions of power and/or PLF of visual γ in SZ (12, 13, 15–18, 20), other studies have not found deficits (14, 19), and increased γ has been reported in SZ (58) as well as schizotypal individuals (59). The factors responsible for the variance in the reported findings are not yet clear, but it can be concluded that there is not a general deficiency of γ generation in the visual cortex in schizophrenia.

Some limitations of this study are clear. One issue is that the SZ had been taking antipsychotic medications for many years, so the degree to which antipsychotics may have influenced the findings is unknown, despite our efforts to assess such possible effects by correlating with chlorpromazine equivalents. Another is that small effects of attention on γ oscillations may not have been detectable due to the sample sizes used here.

This study demonstrates that attentional load effects are manifested in healthy individuals as β activity over areas involved in attentional control and stimulus representation. Individuals with chronic schizophrenia, however, show abnormal β attentional load effects, suggesting that β-generating circuits may constitute part of the neural substrates underlying the cognitive control deficits that figure prominently in this disorder (60, 61). We note that there is also prior evidence for β oscillation abnormalities in SZ: Uhlhaas et al. (19) reported evidence of reduced inter-regional β synchronization during Gestalt perception, and Krishnan et al. (13) found reduced visual steady-state responses to β frequency stimulation. Therefore, the investigation of β oscillations may provide new insights into the neural circuit abnormalities that underlie schizophrenia.

## Conflict of Interest Statement

The authors declare that the research was conducted in the absence of any commercial or financial relationships that could be construed as a potential conflict of interest.
